# Molecular Characterization and Chromosomal Distribution of a Species-Specific Transcribed Centromeric Satellite Repeat from the Olive Fruit Fly, *Bactrocera oleae*

**DOI:** 10.1371/journal.pone.0079393

**Published:** 2013-11-14

**Authors:** Konstantina T. Tsoumani, Elena Drosopoulou, Penelope Mavragani-Tsipidou, Kostas D. Mathiopoulos

**Affiliations:** 1 Department of Biochemistry and Biotechnology, University of Thessaly, Larissa, Greece; 2 Department of Genetics, Development and Molecular Biology, Aristotle University of Thessaloniki (AUTH), Thessaloniki, Greece; Florida State University, United States of America

## Abstract

Satellite repetitive sequences that accumulate in the heterochromatin consist a large fraction of a genome and due to their properties are suggested to be implicated in centromere function. Current knowledge of heterochromatic regions of *Bactrocera oleae* genome, the major pest of the olive tree, is practically nonexistent. In our effort to explore the repetitive DNA portion of *B. oleae* genome, a novel satellite sequence designated BoR300 was isolated and cloned. The present study describes the genomic organization, abundance and chromosomal distribution of BoR300 which is organized in tandem, forming arrays of 298 bp-long monomers. Sequence analysis showed an AT content of 60.4%, a CENP-B like-motif and a high curvature value based on predictive models. Comparative analysis among randomly selected monomers demonstrated a high degree of sequence homogeneity (88% – 97%) of BoR300 repeats, which are present at approximately 3,000 copies per haploid genome accounting for about 0.28% of the total genomic DNA, based on two independent qPCR approaches. In addition, expression of the repeat was also confirmed through RT-PCR, by which BoR300 transcripts were detected in both sexes. Fluorescence *in situ* hybridization (FISH) of BoR300 on mitotic metaphases and polytene chromosomes revealed signals to the centromeres of two out of the six chromosomes which indicated a chromosome-specific centromeric localization. Moreover, BoR300 is not conserved in the closely related Bactrocera species tested and it is also absent in other dipterans, but it’s rather restricted to the *B. oleae* genome. This feature of species-specificity attributed to BoR300 satellite makes it a good candidate as an identification probe of the insect among its relatives at early development stages.

## Introduction

Repetitive DNA elements constitute a significant portion of eukaryotic genomes. According to their genome organization they can be grouped into interspersed sequences and those arranged in tandem, including satellite DNAs. The latter category is comprised of hundreds or thousands of repeats located adjacently to each other forming arrays of the monomeric unit. Such repeats are usually located in the heterochromatin of subtelomeric or centromeric regions of chromosomes [1].

It is generally known that repetitive sequences evolve more rapidly than the rest of the genome by means of concerted evolution. According to theory, diverse homogenization and fixation of the sequences within a genome is achieved as a consequence of molecular drive process [2], [3]. Different rates of homogenization can lead to significant quantitative and qualitative variability (e.g. sequence, copy number, distribution) among closely related species [4]. Accumulation of nucleotide changes in a repeat family results in sequence heterogeneity. This heterogeneity brings about the generation of different satellite DNAs in the same species or among closely related species, due to unequal spread of the mutations between chromosomes [3] or extensive sequence rearrangements. By these processes they can be created either chromosome-specific profiles with particular compartmentalization [5], [6] or a novel repeat restricted to a species [7]. Fluctuation in copy number of satellite DNA due to expansions or contractions of the arrays can also result in species-specific sequences, as was experimentally demonstrated in a study of satellite profiles within the insect genus Palorus [8]. The absence of conservation of these features within taxonomic groups renders satellite DNA markers informative in species discrimination or assessing phylogenetic relationships as in Drosophila species [9], [10] and red flour beetle [11].

Functional roles of the satellite repeats have not yet been attributed by direct experimental evidence, although it is suggested that centromeric satellite DNA contributes to the centromere function [12]. Functional significance and possible mechanisms of action of satellite DNA have been deducted recently (reviewed in [13]. They are thought to interact specifically with DNA-binding proteins to induce epigenetic modifications [14], to be associated with the formation and maintenance of heterochromatin structure and to affect the chromosomal dynamics and genome plasticity ([15] and references therein).

Information on satellite sequences in insects is largely insufficient, despite the vast knowledge accumulated regarding, primarily, to the coding sequences and, secondarily, to other euchromatic parts of the genomes [16]. Intensified research on molecular and genetic level has focused in the last decades on the olive fruit fly, *Bactrocera oleae*, the most devastating pest of olive cultivation worldwide, in an effort to develop strategies of management and biological control ([17]–[19] and references therein, for review [20]). The present study describes the genomic organization, abundance and chromosomal distribution of a novel repetitive sequence of *B. oleae*, designated BoR300, which constitutes part of our attempt to explore the repetitive DNA fraction of the species’ genome.

## Materials and Methods

### Screening of Genomic Library

Screening of an adult olive fly library in λ DASH II [21] was performed on preselected library fractions, as described in [22]. The probe used for the screening was a ∼400 bp PCR product of a retrotransposon fragment (Tsoumani et al. unpublished data) after labeling with 11-dUTP-biotin by random priming (DecaLabel™ DNA Labeling Kit, Fermentas, Burlington, Canada) at a hybridization temperature of 65°C. The probe amplification was carried out in a 20 µl PCR reaction volume using 1.5 mM MgCl_2_, 1× PCR reaction buffer, 1 unit Taq DNA polymerase (Bioline, London, UK), 0.4 pmol of each forward and reverse primers and 0.8 mM dNTPs. The amplification conditions were as follows: 94°C 4 min; 94°C 30 s, 47°C 30 s, 72°C 30 s for 30 cycles; 72°C 5 min, using the forward primer (5′-AGTGTTCTGATCAATGGC-3′) and the reverse (5′-CAGCATCAGGTAGTGTCG-3′).

### Unidirectional Deletions of Plasmid DNA

Plasmid subclones of the cloned 8,000 bp fragment were produced by a set of nested unidirectional deletions with the use of exonuclease III (ExoIII) [23]. The protocol used is described in [24] with minor modifications. In brief, the recombinant plasmid was double digested with the restriction endonucleases BamHI and PstI. The recovered DNA after phenol/chloroform purification was dissolved in 45 µl ExoIII buffer and subsequently digested with ExoIII (300 u) for the generation of unidirectional deletions. Digestion proceeded at about 210 bp/min and 2.5 µl samples were removed at 2 min intervals. Subsequently, the 20 time-point harvested samples were treated with S1 nuclease and 4 µl aliquots of each time point sample were electrophorized to determine the extent of the digestion. Four samples of the desired size were pooled, blunt-ended with Klenow, recircularized with T4 DNA ligase and used to transform DH5α *Escherichia coli* cells. Plasmids with deleted inserts were identified by gel electrophoresis of mini-preparations of DNA using the Promega Wizard Plus Minipreps DNA Purification System according to the supplier’s instructions. DNA sequencing of randomly selected inserts was performed by Macrogen Inc (Korea) using the universal M13 forward and reverse primers.

### Cloning and Sequence Analysis

*In silico* analyses for restriction sites and repeat motifs search were performed using the Omiga software (Kramer 2001), sequence alignments using the ClustalW online software [25], whereas homology searches were performed with BLAST programs available on NCBI [26]. The prominent band (∼ 300 bp) of a HaeIII restriction fragment of the ExoIII subclone pExo34 was gel purified by the Wizard1 SV Gel and PCR Clean-Up System (Promega, Madison, WI, USA) following the manufacturer’s instructions, ligated into the plasmid vector pBlueScript-SK(+) with EcoRV blunt termini and finally used to transform competent *E. coli* DH5α cells according to standard procedures (Sambrook et al. 1989). The recombinant plasmid DNA was finally isolated with the use of the Promega Wizard Plus Minipreps DNA Purification System according to the supplier’s instructions. The curvature-propensity plot was calculated with DNase I parameters of the bend.it server (http://www2.icgeb.trieste.it/dna/bendit.html) according to [27]. The values of the predicted curvature are presented as the deflection angle per 10.5 residue helical turn (1°/bp).

### Nucleotide Sequence Accession Numbers

Sequence data have been submitted to GenBank under the following accession numbers: KF680582– KF680589.

### Fly Samples and DNA Isolation

*B. oleae* and *C. capitata* genomic DNA was extracted from pooled adult flies of the ‘Demokritos’ and ‘Benakeion’ strains, respectively, maintained in our laboratory. The Canton-S (Canton Special) wild-type strain of *D. melanogaster* was used as a source for the Drosophila DNA. Genomic DNA was isolated using the Wizard Genomic DNA extraction kit (Promega, Madison, WI, USA) and quantified spectrophotometrically.

### Southern Blot Analysis of Digested Genomic DNA

Four µg genomic DNA samples of different fly species were digested with the HaeIII restriction endonuclease, separated on 1% agarose gels and transferred onto Hybond-N+ nylon membranes (Amersham Biosciences) using alkaline transfer. Southern hybridization was performed according to standard protocols described by Sambrook et al. (1989) at 60°C using 20 ng/ml of labeled probe. The cloned monomer of the satellite (BoR300) was used as probe after labeling with biotin-11-dUTP using a random primer DNA labeling kit (Fermentas, Burlington, Canada).

### Real-time qPCR Using SYBR Green I Dye

Real time-PCR (qPCR) reactions were carried out in a total volume of 20 µl consisting of 1 µl of template DNA, 1× of qPCR master mix and 150 nM of each primer (Table 1). The primers used (BoR300F and BoR300R) were designed with opposite orientation in order to amplify tandemly arranged monomer units. The thermal cycling conditions were as follows: 95°C 10 min, 95°C 10 s, 53°C 10 s, 72°C 10 s for 40 cycles. The fluorescence signal for SYBR Green I dye was automatically measured for both standards and unknowns at the end of each extension step at 72°C in the same run. At the end of each qPCR assay, a melting curve ramp in the default thermal profile was performed to control the amplification specificity. The qPCR products were further confirmed by electrophoresis. Real-time PCR was undertaken using the KAPA™ SYBR® Green FAST qPCR kit (KapaBiosystems, Boston, MA) and the Mx3005P spectro-fluorometric thermal cycler operated by the MxPro™ PCR software (Stratagene). The Ct values for each reaction were calculated automatically by the software, by determining the PCR cycle number at which the reporter fluorescence exceeded background. Triplicate reactions were conducted in each assay, and each assay was repeated twice. The genomic samples were determined by three replicates in each experiment. No template control (NTC) was also included in each experimental run as negative control to verify that no reagent contamination had occurred by the target DNA.

**Table 1:** Primer sequences and parameters of the RT-PCR and qPCR assay.

Target sequence	Primer	Primer sequence (5′→3′)	Ta (°C)	Amplicon size (bp)
***ace***	Boace2F	TTCGCGTCAATACAGTGTCG	53	315
	Boace2R	CTTTCTTGCACACAGGTTGC		
**BoR300**	BoR300F	TGCACATATGCATCTACC	53	298
	BoR300R	CTTAAATGGGTTGAACG		
**BoEST_175**	epic175F	AAAATGCGCTTCCATAAGATCG	53	420
	epic175F	ATCCAACATCCTTGGAATATCG		

In order to assess BoR300 copy number in the olive fly genome, two different methods were followed: i) the relative and ii) the absolute qPCR based approaches [28], [29].

#### i) Relative qPCR

The repeat copy number is determined by comparing the Ct (threshold cycle) value of the target sequence with the respective Ct value of a single copy reference gene [29] using the genomic DNA template. Serial dilutions (10 pg, 100 pg, 1 ng) of the genomic DNA were used for each primer pair used. The efficiency of qPCR amplification (E) was determined by the slope of each standard curve, as follows: 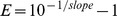. Copy number at the relative approach was calculated using the equation: 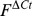. F is the amplification factor of the qPCR amplicon which equals 2 if the amplification efficiency is 100%, which means that amplicons double every cycle during the exponential phase of the PCR. ΔCt is the difference in mean Ct (threshold cycle) value of the amplicon targeting *ace* (single-copy reference standard for these studies) and mean Ct value of the amplicon whose copy number is being estimated (BoR300 repeats).

#### ii) Absolute qPCR

The absolute quantity of the repeat in the genomic DNA is obtained by interpolating the Ct value of the target sequence against the standard curve generated by the dilution series of a standard plasmid. Each PCR reaction was performed using as template either cloned repeats (standards) or genomic DNA (unknowns). Initially, a series of the recombinant plasmid p276-1 dilutions were prepared (0.06 fg, 0.6 fg and 60 fg) and the copy number of BoR300 in each dilution was calculated based on the equation [30]:
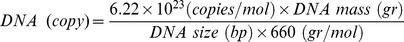



Subsequently, the Ct values measured by the qPCR for each dilution of the p276-1 automatically generated the standard curve (measured Ct values against BoR300 copies). Finally, the repeat copies in the unknown genomic DNA sample (10 pg) were determined by interpolating its Ct value against the logarithm of their initial template copy numbers of the standard curve.

### RT-PCR Analysis

Total RNA was extracted from pooled adult *B. oleae* flies (from the “Demokritos” laboratory strain) using TRIsure-reagent (Bioline, London, UK) according to the manufacturer's instructions and subsequently treated with TURBO DNA-free DNase (Ambion®, USA) to remove any co-extracted genomic DNA. 1 µg of total RNA was reverse-transcribed using random primers with the MMLV Reverse Transcriptase (GeneON, Germany) according to the manufacturer’s instructions. One-tenth of the first- strand cDNA was further used for standard PCR with primers BoR300F and BoR300R, as well as epic175F and epic175R. The PCR was carried out as described above and the amplification profile was: 94°C 4 min; 94°C 30 s, 52°C 30 s, 72°C 20 s for 30 cycles; 72°C 5 min. Primers, annealing conditions and expected product sizes are given in Table 1. All PCR products were resolved in 1% agarose gels stained with ethidium bromide.

### Chromosome Preparations and Fluorescence *in situ* Hybridization

Spread preparations of mitotic and polytene chromosomes were made from the brain (cerebral ganglia) and the salivary glands, respectively, of third instar larvae and young pupae (1–2 days old) following the methods described in detail by [19]. For FISH, the BoR300 monomer cloned in pBS-SK(+) was used as probe. Labeling was performed by PCR using the universal M13 forward and reverse primers (Invitrogen, Life Technologies Inc., San Diego, CA, USA) and biotin-11-dUTP (ROCHE) as precursor. Pretreatment of chromosome preparations, hybridization, detection and image analysis was performed as described in [19].

## Results and Discussion

### Isolation and Sequence Analysis of the Repeat

**Figure 1:**
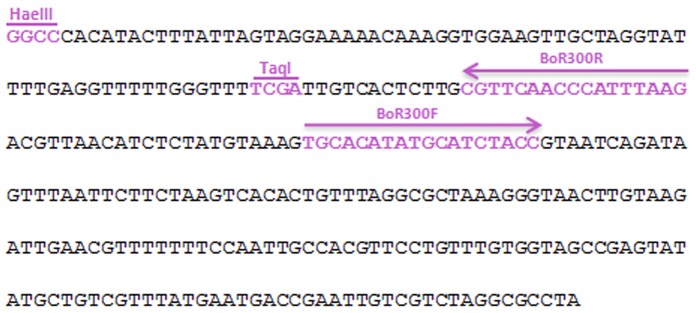
Nucleotide sequence of the monomer BoR300. The arrows indicate the outfacing primer pair: the reverse (BoR300-R) and the forward (BoR300-F) primer respectively. The restriction sites of the restriction endonucleases HaeIII and TaqI are also highlighted.

**Figure 2:**
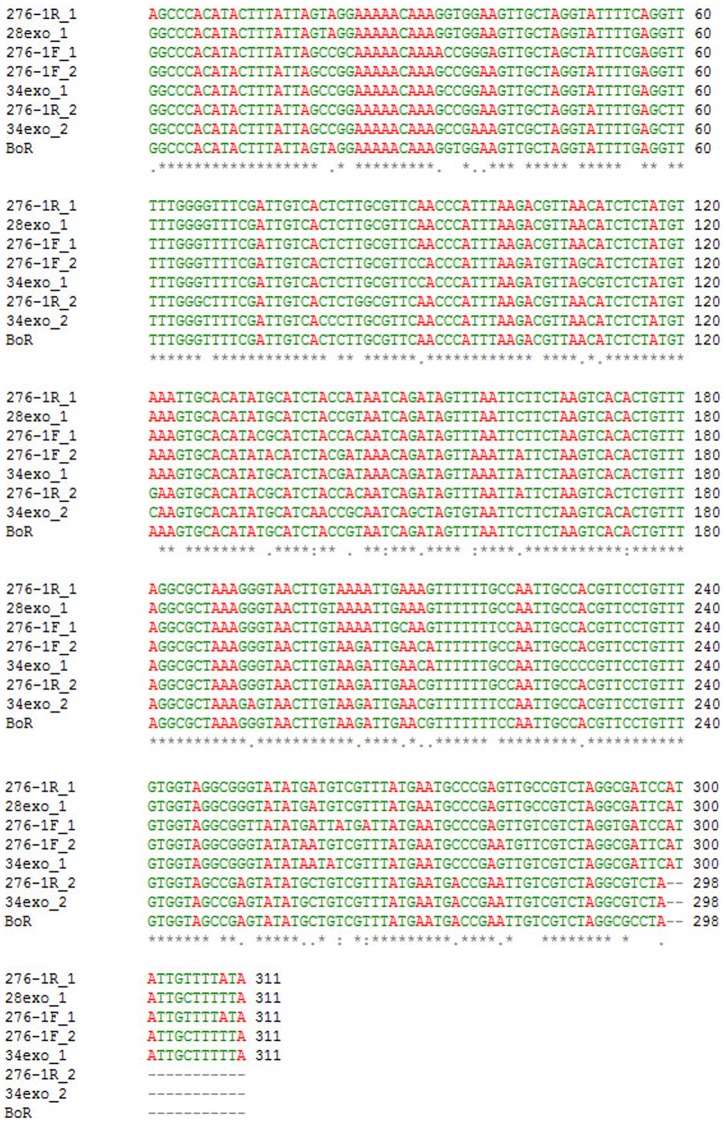
Variability of BoR300. Multiple sequence alignment of different BoR300 units obtained from the terminal sequence reads of the original 8-kb p276-1 clone and two internal subclones (pExo34, pExo28) derived from ExoIII serial deletions of p276-1. In addition, a HaeIII cloned BoR300 unit (originated from clone pExo34) is also included. The corresponding name-code of each repeat and the respective subclone of its origin were as follows: i) right end of p276-1∶276-1F_1 and 276-1F_2, ii) left end of p276-1∶276-1R_1 and 276-1R_2, iii) left end of the subclone pExo34∶34exo_1 and 34exo_2, iv) left end of the subclone pExo28∶28exo_1, v) sequence of the BoR300 unit: BoR300.

In our effort to obtain the full-length sequence of a new *Bactrocera oleae* retrotransposon distributed primarily in heterochromatic regions (Tsoumani et al. unpublished data), extended sequence analysis of various genomic library phage clones was performed. A phage EcoRI restriction fragment of ∼8,000 bp was gel purified, cloned into pUC19 and sequenced. Both the sequencing data of the forward and reverse ends of the plasmid designated as p276-1 revealed the presence of a 298 bp tandem repeat (Figure 1). This observation was indicative that along its entire length the repeat was organized in tandem. The homology search yielded no significant results of similarity with known sequences deposited in current databases. The monomer length of the sequence (GenBank KF680582) is 298 bp with an AT content of 60.4%. Dot plot analysis did not reveal any significant direct or inverted subrepeats, indicating that the satellite is unique and has not been derived from the amplification of simpler motifs. Efforts to subclone the 8.0 kb sequence in smaller restriction fragments failed, probably due to the absence of common restriction sites or the presence of particular secondary structures. To overcome this difficulty, we generated a series of ExoIII deletion clones of the desired insert length, which were subsequently partially end-sequenced. As expected, all analyzed samples contained the repeat motif in tandem arrays with a typical head-to-tail orientation. Pairwise alignments among the available repeat monomers yielded high identity scores. The sequence similarity ranged from 88% – 97% (Figure 2), which falls within the limits generally reported in insects, according to which the intra-specific variability of such sequences ranges from 1% to 13% [31]. The different evolutionary rate along the entire sequence, suggests that selective forces act to keep the monomers homogenized, rather than independent mutational events result in sequence variants.

**Figure 3:**
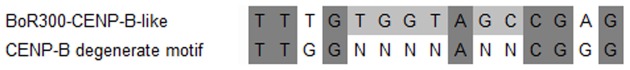
CENP-B like motif. Comparison of the CENP-B like motif (237–252 bp) found in BoR300 with the degenerate motif considered to bind the CENP-B protein.

Further sequence inspection revealed a region of considerable homology to the consensus sequence for the CENP-B box. The CENP-B box has been identified within satDNA of mammals and insects [32], [33]. It is a conserved 17 bp motif in human α-satDNA and a binding site for centromere protein B (CENP-B). The BoR300 CENP-B-like motif, that was identified in region 223–219 nt (Figure 3), includes 7 out of 9 conserved nucleotides of the degenerate mammalian CENP-B box. This region indicates a putative binding site of the corresponding *B. oleae* centromere-associated proteins. The conservation of this motif between species perhaps comes out of the necessity to maintain satellites’ interaction with these proteins, even though the nature of these associations is not well understood.

**Figure 4:**
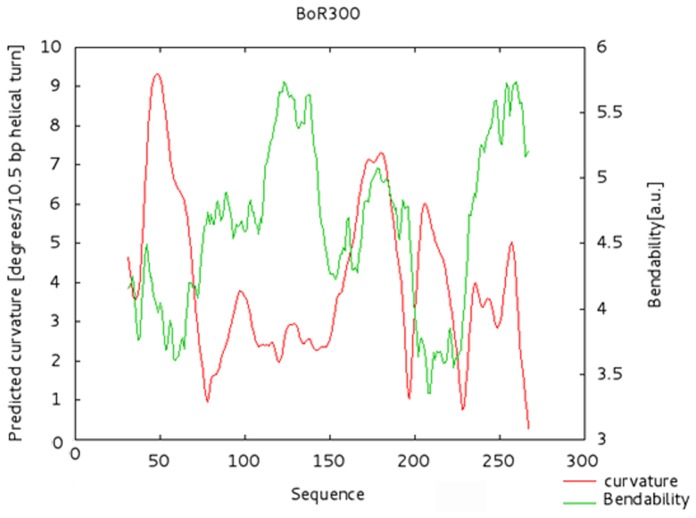
Curvature analysis of BoR300. Curvature-propensity plot of the consensus sequence of BoR300 showing that the maximum peak is located at the beginning of the monomer (red line).

*In silico* prediction of the curvature and bendability was also performed based on a model of sequence-dependent DNA bending. The histogram of the curvature-propensity plot, calculated with DNase I parameters of the bend.it server, presented a region near 50 bp with a high curvature propensity value of about 10°/helical turn (Figure 4), which lies within the range (5–25°/helical turn) of experimentally tested curved motifs. This property suggests a possible curved conformation for BoR300. Moreover, two moderate peaks near 170 and 200 bp of about 6 and 7°/helical turn were also observed. All curvature profiles revealed by the predictive model correspond to DNA regions with AT-rich tracts, as was also affirmed by their respective low GC content plot. The propensity for bending and twisting is a conserved feature of satellite DNA that is proposed to contribute to centromeric heterochromatin formation. In insects, however, this feature is constantly maintained even among non-centromeric satellite DNAs. Even if the exact implications of DNA curvature in centromere organization are not well established, many reports claim that these particular structural DNA features might play an essential regulatory role in DNA–protein interplay, which are necessary for the tight packing of the heterochromatin and consequently in the structural stability and compaction of centromeric regions [34].

### Transcription of BoR300

**Figure 5:**
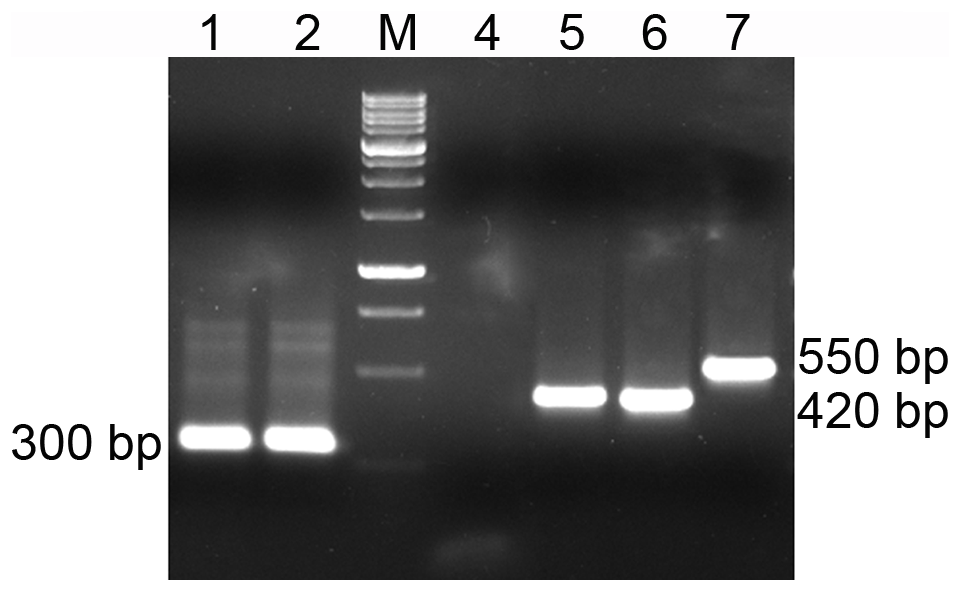
Analysis of BoR300 transcription. Total RNA from male (lane 1) and female (lane 2) adult *B. oleae* flies were extracted and reverse-transcribed using random oligonucleotides. Satellite transcripts were amplified by PCR using BoR300-F and BoR300-R primers. M represents the molecular marker (1000 bp/1 kb BLUE DNA Ladder, GeneON). The epic175F and epic175R primers were also used, to check the presence of any DNA contamination on both male and female cDNAs (lanes 4 & 5 respectively). The amplification results were compared to those obtained using the genomic DNA as template (lane 7) at which the product size was 550 bp.

Transcription of the BoR300 satellite was assayed by reverse transcription with satellite-specific oligonucleotides on RNA extracted from both sexes of adult *B. oleae* flies. Amplification of the transcripts was demonstrated for all cDNA samples, corresponding mainly to monomers, but also to fainter bands of multimers of the satellite RNA (Figure 5). No sex-specific transcripts were observed between males and females, indicating the absence of gender-specific expression. The amplification products were real BoR300 transcripts and could not have resulted from the presence of DNA in the RNA preparation, since the RNAs were treated with DNase prior to the reverse transcription in order to remove any co-extracted DNA. To confirm this claim, an additional EPIC (Exon Primed-Intron Crossing) PCR was carried out as control for DNA contamination, using primers that were flanking an intron [17]. As a result, products of reduced size were amplified revealing the absence of the intron in each cDNA template comparing with the genomic DNA template.

The transcription of satellite DNA seems to be a generally occurring phenomenon described in many different organisms including vertebrates, invertebrates and plants. In particular, insect satellite transcripts have been differentially detected in association with development, differentiation and stress response [35]. In Diptera, transcription proceeds from both DNA strands by read-through either from upstream genes or transposable element promoters, or from promoters and transcription initiation sites within the repeat sequence [36] and is reported to be under the control of RNA interference machinery [37]. The satellites that act as precursors of small interfering RNAs operate to maintain the silenced state of centromeric and pericentromeric repeats. However their functional significance and the molecular mechanism of transcription are still complex to explain at present (for review see [35], [38]). The most dominant view suggests a regulatory role of satellite transcription in chromatin epigenetic modulation and the post-transcriptional expression control of genes that contain repeat-complementary regions [13].

### Chromosomal Distribution

**Figure 6:**
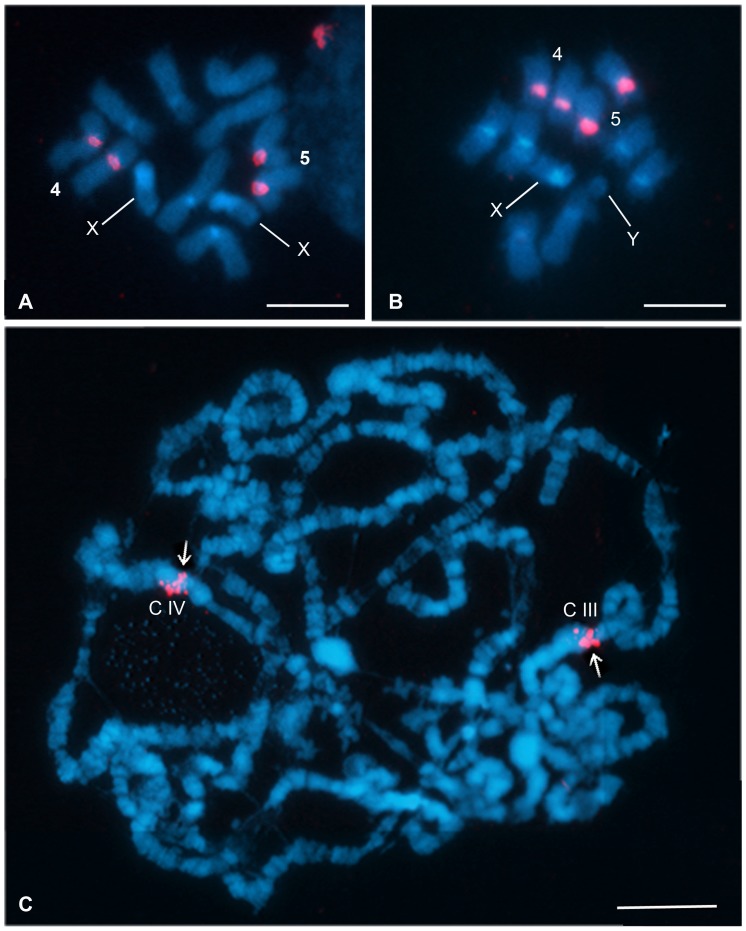
FISH with the BoR300 probe on mitotic and polytene nuclei of *Bactrocera oleae.* Chromosomes were counterstained with DAPI (blue). Female (a) and male (b) metaphase showing strong hybridization signals (red) on the centromeres of chromosomes 4 and 5. Polytene complement (c) showing strong hybridization signals (red) on the centromeric heterochromatic blocks (C) of chromosomes III and IV (arrows). Bar  = 3 µm (a, b), 20 µm (c).

Ιn order to assess the chromosomal distribution of the BoR300 repeat, fluorescence *in situ* hybridization (FISH) was carried out to both mitotic and polytene complements of *B. oleae* using the repeat unit as probe. In mitotic metaphases the probe hybridized exclusively to the centromeres of two autosome pairs, namely 4 and 5, while in polytene nuclei it was found to be associated with the centromeric areas of polytene chromosomes III and IV (Figure 6). No additional signals were observed either at the mitotic or the polytene spreads.

The cytogenetic data of *B. oleae* is well established. Its mitotic karyotype has a diploid set of 2n = 12 chromosomes consisting of a pair of heteromorphic sex chromosomes (pair 1) and five autosomes (pairs 2–6), while its polytene complement consists of five banded chromosomes (I-V) corresponding to the autosomes of the mitotic complement and a heterochromatic mass representing the sex chromosomes [19], [20], [39], [40]. Since the nomenclature of the mitotic and polytene elements was based exclusively on their relative size in descending order, no correlation between the two chromosome sets exists [39]. A large number of ESTs [17], microsatellites [41] and other molecular markers [42], [43] have been mapped on *B. oleae* polytene chromosomes by *in situ* hybridization. However, none of them proved to be informative on mitotic chromosomes. This is due to the fact that polytenization is a result of several rounds of euchromatin replication without separation of chromatids. Consequently, the sensitivity of signal detection on polytene chromosomes is increased several times, making it possible to map single copy targets on polytene chromosomes, a nearly impossible task for mitotic spreads. However, multiple copy targets, such as the repetitive 18S rRNA genes [44] and satellite repeats (present study), can be localized effectively on both complements.

The successful hybridization of the BoR300 sequence on both *B. oleae* mitotic and polytene chromosomes allowed the direct correspondence of two autosomes between the two complements, namely the 4 and 5 mitotic pair with the III and IV polytene elements. Even though it was not feasible to discriminate the one-to-one correspondence between them because of the similar length of these chromosomes in both sets and the absence of any additional marker [20], cytological mapping reported here constitute the first correlation between two autosomes of the two types of chromosomal complements: mitotic and polytene. Moreover, the specific hybridization of BoR300 repeats exclusively at the heterochromatic centromeric areas of the *B.oleae* genome indicates the absence of these repeats along the arms of the chromosomes. Such specific distribution pattern of repetitive elements is not unusual, as chromosomal specificity has been previously reported in other species as well. In Drosophila, for instance, different repeated sequences have been identified in each centromeric region [5], [45]. It has been suggested that turn over mechanisms may be responsible for the creation of chromosome specific satellite families through the unequal spread of mutations and the inducement of extensive rearrangements which can lead to the generation of novel repeat variants [3], [4].

### Genomic Content of the Satellite DNA

**Figure 7:**
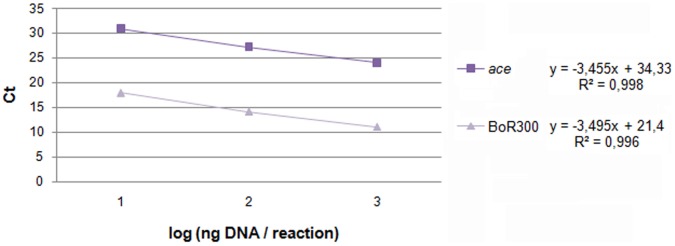
Standard curves for the reference single copy gene (*ace*) and the repeats of BoR300. The construction of the curves was based on serial 10-fold dilutions of the genomic DNA template used (10 pg, 100 pg, 1 ng). For each amplicon, qPCR determined Ct values were plotted against the logarithm of their initial concentration (1, 2 and 3 values respectively).

The estimation of the repeat copies in the genome was conducted by two independent ways: a relative and an absolute quantification method. For the relative method, two separate qPCR reactions were carried out, one amplifying BoR300 repeats and the other the single-copy acetylcholinesterase (*ace*) gene, which served as a single-copy control target for the genomic DNA template. The threshold cycle value, Ct, of the sequence of interest (BoR300 repeat) was compared to the Ct value of the single-copy reference gene (Figure 7). The difference in Ct values was then used to derive the copy number. The amplification factors (F) for the unknown and the control amplicons were estimated as 93.4% and 94.7% respectively. The almost equal values indicate that the amplification kinetics between the reactions were similar, allowing valid quantitative comparisons. The results from the relative quantification are shown in Table 2, according which the calculated copy number was 3,530 per haploid genome. Given the estimated *B. oleae* genome size of about 322 Mb or 0.352 pg [46] and the length of the repeat unit of 298 bp, the relative qPCR approach showed that the repeats constitute approximately 0.32% (1.062 Mb) of the *B. oleae* genome.

**Table 2:** Comparative analysis data of the qPCR amplification curves of BoR300 repeats and the single copy gene (*ace*), for the absolute estimation of BoR300 copies.

	Intercept^a^	Slope^b^	Efficiency (E)^c^	ΔCt^d^	F^ΔCte^
**BoR300**	34.33	3.455	93.4%	12.93	0.353×10^4^
***ace***	21.4	3.495	94.7%		

a,b Intercept and slope of the linear regression lines (Figure 6).

c The efficiency of the reaction was estimated by the equation: E = 10^−1/slope^-1.

d ΔCt value is obtained by comparing the y-axis intercepts derived from the two lines-of-best-fit of the target sequences BoR300 and *ace*.

e According to the mean efficiency of the reactions (94.05%) the amplification factor F was 1.881.

In the second approach, absolute quantification determined the exact copy concentration of repeat sequence by relating the Ct value to a standard curve. For that purpose, qPCR reactions were conducted with two different template sources: the recombinant plasmid p276-1 (with a known number of BoR300 repeats) and the genomic DNA (with unknown number of BoR300 repeats). The copy number of BoR300 within genome was estimated by interpolating the Ct value of the genomic DNA sample to the generated standard curve of the plasmid p276-1 copies, which was finally calculated as 2,661 copies (Table 3). According to this measured number the repeat copies were estimated to constitute approximately 0.25% (0.798 Μb) of the *B. oleae* genome.

**Table 3:** qPCR analysis data of the relative estimation of BoR300 repeats using a reference standard curve.

	Sample concentration (pg)^a^	Calibration curve	Repeat copy number^b^	Copy number per haploid genome^c^
**BoR300**	10	y = −3.52×log(x) +35.197	75,610±2,893^d^	2,661

a Initial template concentration of the *B*. *oleae* genomic DNA (pg) used at the qPCR reactions.

b Mean copy number of BoR300 repeats which was estimated for the initial template concentration of the genomic DNA (a) based on the standard curve.

c *B. oleae* haploid genome size: 0.352 pg.

d Standard Error (SE) for the triplicate measurements (n = 3).

The results obtained by both approaches do not differ considerably. The observed difference could be attributed to the general repetitive nature of these sequences and the difficulty of their handling. Their organization in long tandem arrays makes the structural analysis extremely difficult due to their potential secondary conformations, which could hinder the precise estimation of their copy number in a genome. It should be noted that the structure of the cloned sequence (p276-1) used as a standard in the absolute method, might have contributed to a less efficient primer hybridization. Although the available monomer sequences derived from this clone did not reveal the presence of any significant inverted or palindromic subrepeats or even sequences inserted within the array, this could not be excluded to occur to some variants which might have finally led to an underestimation of the calculated copies. However, the estimated copy number of the repeat units is in agreement with several previously reported studies of related species concerning the genomic content of satellite sequences. The 44 bp pericentromeric repeat of its close relative *C. capitata*, which was also proved species-specific with selective chromosomal distribution, was estimated to represent about 0.24% of the genome [47].

### Species Specificity of the Repeat

**Figure 8:**
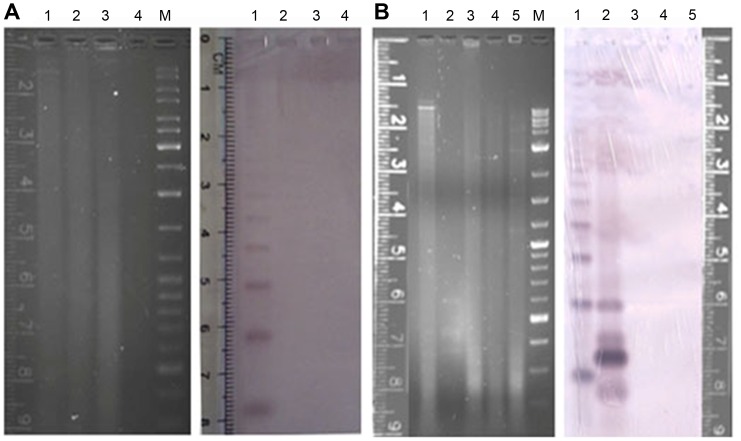
Species distribution of BoR300 repeats. HaeIII-digested genomic DNA of representative species of the Diptera family and the genus Bactrocera was analyzed by Southern hybridization using as probe the biotinylated monomer (298 bp) of the repeat. (**A**) Southern blot analysis of digested genomic DNA from the following dipteran species: *B. oleae* (lane 1), *C. capitata* (lane 2), *D. melanogaster* (lane 3) and *An. gambiae* (lane 4). **B**) Southern blot analysis of digested genomic DNA from the following Bactrocera species: *B. oleae* (lane 1), *B. correcta* (lane 3), *B. cucurbitae* (lane 4), and *B. dorsalis* (lane 5). In Lane 2 is the cloned monomer of the repeat. M represents the molecular marker (SM0331, Fermentas).

The presence of the BoR300 repeats in the Tephritid family and other dipteran species (*D. melanogaster*, *An. gambiae*) was investigated by Southern hybridization of HaeIII-digested genomic DNA. The Southern hybridization was carried out by using the cloned PCR amplified repeat monomer as probe. The analysis revealed the presence of a regular satellite ladder pattern in the *B. oleae* genome, indicating a tandem arrange of the BoR300 repeat in the olive fly genome. No hybridization signal was obtained in any of the other species tested within the limits of the sensitivity of the Southern hybridization (Figure 8). Therefore, blot results have clearly revealed that the BoR300 repeat is restricted to *B. oleae* genome. Specificity was also assessed by PCR amplification of the basic repeat unit in four additional Bactrocera species (*B. dorsalis, B. invadens, B. correcta* and *B. cucurbitae*). No amplification products were obtained in any other species, confirming the species specificity of the repeat. The fact that BoR300 repeats were not detectable by PCR in related species, excludes the possibility that are present in very low copy number. Its absence from the phylogenetically related species suggests that BoR300 was probably amplified after *B. oleae*’s speciation during the evolution of the Tephritid family. As reported in the literature, the evolution of a repeat within a species may be the consequence of concurrent or independent changes in sequence and/or copy number of the repeat (for a review see [4]) which, in turn, has the potential to influence the evolution of the species. Both types of changes are ruled by the same mechanism of concerted evolution.

This specificity also renders BoR300 a species and chromosome specific molecular marker. Given the fact that several Tephritid species are morphologically indistinguishable at the larval and pupal stages, such tools could be very useful in species identification from soil samples.

## Conclusion

Current knowledge of heterochromatic regions of Tephritid genomes is practically nonexistent. The olive fly is not an exemption. The sequences comprising the centromeres of *B. oleae* are totally unexplored, mainly due to the different focus that *B. oleae* research always has had. Only some repetitive interchromosomal duplications have been reported that appeared to have accumulated to the heterochromatic Y chromosome [18].

In the present study a novel repeat sequence named BoR300 was cloned and further analyzed with regard to its structural and cytological organization. Its structural constraints (sequence length, base content, curvature, as well as abundance and distribution) indicated a satellite repeat. Additionally, its transcriptional activity may indicate involvement in regulation of chromatin organization, possibly through RNAi mechanisms [35]. BoR300 species- as well as chromosome-specificity point towards the satellite’s participation in major genomic or chromosomal rearrangements that characterize the evolution of the species and two of its six chromosomes (mitotic 4 and 5 or polytene III and IV). Given the functional involvement of satellites in chromosome dynamics and their evolutionarily versatile nature, further investigation is needed to clarify the exact role of the repeat.

From an applied point of view, BoR300 could provide an effective molecular tool for species-recognition at specific developmental stages, when it is hard to distinguish between individuals of closely related species. In this regard, in areas where different fruiting trees coexist that may be infested with various Tephritids, such diagnostic probes could successfully determine the type of infestation.

Finally, it is clear that understanding the elusive nature of the repetitive sequences of a genome is a desirable requirement for the subsequent in depth speculation of its organizational structure and function. Although this was beyond the scope of our study, the present results could contribute to future detailed knowledge of repetitive sequences, since repeat libraries are not available in Tephritids. This will therefore enable screening and masking of these repeats, a step of crucial importance when dealing with sequencing and assembly genome projects, in order to reconstruct contigs and to eliminate spurious homology predictions.
